# Supplementary material for: Capacitive in-sensor tactile computing
Source: Nat Commun. 2025 Jul 1;16:5691. doi: 10.1038/s41467-025-60703-7 (PMC12217174; doi:10.1038/s41467-025-60703-7)
Supplement: Supplementary file 1 — Supplementary Information [file 41467_2025_60703_MOESM1_ESM.pdf]

Supplementary Information

Capacitive in-sensor tactile computing

Yan Chen[1,2,3], Jie Cao[1], Jie Qiu[1,2,3], Dongzi Yang[1,2], Mengyang Liu[1], Mengru Zhang[1,2], Chenyang Li[1,2,3], Zhongyuan Wu[4], Jie Yu[1], Xumeng Zhang[1], Xianzhe Chen[1], Zhangcheng Huang[1], Enming Song[4], Ming Wang[1,3], Qi Liu[1,3] and Ming Liu[1,3]**

Table of Contents:

I: Supplementary Notes

II: Supplementary Figures and Captions

III: Supplementary Tables

I: Supplementary Notes

1. Operation flow of the capacitive in-sensor computing system

The capacitive in-sensor tactile computing system process based on the capacitive in-sensor tactile computing kernel requires three phases (Φ_1, Φ_2, Φ_3). We take the 3 × 3 capacitive sensor array as an example to explain the operation flow and calculation principle of the system in detail (Supplementary Fig. 6). For simplicity, all capacitive pressure sensors (C_1 to C_9) in the array have the same initial capacitance value of 0.1 nF. The turn-on time for all T1, T2 and T3 switches is set to 5 ms. The switching interval between T1, T2 and T3 is set to 2 ms. The interval time between the Φ_3 phase and the next Φ_1 phase is set to 42 ms.

At the Φ_1 phase, T3 switch turns on, while all T1 and T2 switches keep off states (Supplementary Fig. 6a). This process is referred to as a refresh operation, which aims to completely clear the charges possibly stored on the readout capacitor C_0. At the Φ_2 phase, nine capacitive pressure sensors are subjected to a variety of tactile stimuli. Then, nine T1 switches turn on, while nine T2 switches and T3 switch keep off states (Supplementary Fig. 6b). The resultant amount of charges (Q_i) stored on each sensor pixel in the array during the charging phase can be calculated as:

$$Q_i = C_i \times V_i, \quad i=1,2\ldots9 \tag{1}$$

At the Φ_3 phase, all nine T1 switches turn off simultaneously and then the nine T2 switches turn on (Supplementary Fig. 6c). This initiates a charge sharing process

where individual charges (Q_i) stored on each pressure sensor pixel will converge to form a summed charge (Q_{sum}).

$$Q_{sum}=\sum_{i=1}^{9} Q_i=\sum_{i=1}^{9} C_i \times V_i\,, \quad i=1,2\ldots 9 \tag{2}$$

The summed Q_{sum} is then read via the fixed capacitor C_0, which is connected in parallel with the pressure sensor pixels in the array. Since the capacitor C_0 is connected in parallel, a portion of the summed Q_{sum} will flow to the capacitor C_0, resulting a peak voltage value (V_{out}) on C_0. The charges stored on the capacitor C_0 can be accurately read by its peak voltage value (V_{out}), which can be expressed as:

$$V_{out}=\frac{Q_{sum}}{\sum_{i=1}^{9} C_i+C_0}=\frac{\sum_{i=1}^{9} C_i \times V_i}{\sum_{i=1}^{9} C_i+C_0}\,, \quad i=1,2\ldots 9 \tag{3}$$

In our implementation, the charges stored on the C_0 capacitor is used to represent the summed Q_{sum}. In other words, V_{out} serves as the final calculated result of capacitive in-sensor tactile computing system. Obviously, a larger C_0 will cause more charges stored on the capacitor C_0, leadning to a high readout accuracy on Q_{sum}. However, a large C_0 will result in an excessively small V_{out} that is unfavorable for reliable sampling. In our case, we selected a fixed capacitor of 10 nF as C_0, which is a trade-off strategy. To easily obtain the calculated result, we selected the moment when the T2 switches turn from on to off states as the sampling point to capture the peak value of V_{out}.

2. Simulated calculation processes of the in-sensor tactile computing system

Even though the capacitive pressure sensor array is not connected to the printed circuit board including switch chips (Supplementary Fig. 5), the V_{out} port still generates a tiny baseline voltage (V_{base}) when the voltage input terminals are biased. The V_{base} results from the leakage current of switch chips and the parasitic capacitance of the printed circuit board system. For noise reduction task, a V_{base} of 0.13 V was observed in the V_{out} when the input voltage biases of the system were set to an identical value of 3.3 V (Supplementary Fig. 10k). For edge detection task, a V_{base} of 0.04 V was observed when the input voltage biases from V_1 to V_9 were set to 1 V, with the exception of V_5 that was set to -8 V (Supplementary Fig. 12r). After considering the effect of leakage current and parasitic capacitance, the simulation calculated V_{out} value of the capacitive in-sensor tactile computing system should be sum of the calculated V_{out} value (obtained from Eq.3) and the corresponding V_{base}, given by:

$$V_{out}=\frac{Q_{sum}}{\sum_{i=1}^{9} C_i+C_0}+V_{base}=\frac{\sum_{i=1}^{9} C_i\times V_i}{\sum_{i=1}^{9} C_i+C_0}+V_{base},\quad i=1,2\ldots9 \qquad (4)$$

During the simulated calculation processes, the capacitive pressure sensors are assumed to have uniform sensing characteristics, with capacitance values of 0.1, 0.54, 0.74, and 0.86 nF at applied pressures of 0, 0.8, 1.6, and 2 kPa, respectively (Fig. 2c). The readout capacitor C_0 is 10 nF.

Calculated processes for noise reduction task (Fig 4b):

For pattern mode 0: With all nine sensors at 0 kPa pressure, the simulation calculated value is:

$$V_{out} = \frac{\sum_{i=1}^{9} C_i \times V_i}{\sum_{i=1}^{9} C_i + C_0} + 0.13\ V = \frac{(0\times0.86\ nF+9\times0.1\ nF)\times3.3\ V}{0\times0.86\ nF+9\times0.1\ nF+10\ nF} + 0.13\ V$$
$$= 0.402\ V$$

For pattern mode 1: With one sensor at 2 kPa and other eight sensors at 0 kPa, the simulation calculated value is:

$$V_{out} = \frac{\sum_{i=1}^{9} C_i \times V_i}{\sum_{i=1}^{9} C_i + C_0} + 0.13\ V = \frac{(1\times0.86\ nF+8\times0.1\ nF)\times3.3\ V}{1\times0.86\ nF+8\times0.1\ nF+10\ nF} + 0.13\ V$$
$$= 0.600\ V$$

Other patterns follow the same calculation method. All the simulation calculated values are listed in the Supplementary Table 2.

Calculated processes for noise reduction task under the mixed tactile input (Fig 5e):

When one sensor is applied by the pressure stimulus of 2 kPa, one sensor is applied by the pressure stimulus of 1.6 kPa, other seven sensors are applied by the pressure stimuli of 0.8 kPa, the simulation calculated value is:

$$V_{out} = \frac{\sum_{i=1}^{9} C_i \times V_i}{\sum_{i=1}^{9} C_i + C_0} + 0.13\ V = \frac{(1\times0.86\ nF+1\times0.74\ nF+7\times0.54\ nF)\times3.3\ V}{1\times0.86\ nF+1\times0.74\ nF+7\times0.54\ nF+10\ nF} + 0.13\ V$$
$$= 1.284\ V$$

Other patterns follow the same calculation method.

Calculated processes for edge detection task (Fig 4e):

For pattern mode A0: With eight sensors at 2 kPa and central sensor at 0 kPa, the simulation calculated value is:

$$V_{out} = \frac{\sum_{i=1}^{9} C_i \times V_i}{\sum_{i=1}^{9} C_i + C_0} + 0.04\ V = \frac{(8\times0.86\ nF)\times1\ V+1\times0.1nF\times(-8)\ V}{8\times0.86\ nF+1\times0.1\ nF+10\ nF} + 0.04\ V$$
$$= 0.398\ V$$

For pattern mode A1: With seven sensors at 2 kPa, one sensor at 0 kPa and central sensor at 0 kPa, the simulation calculated value is:

$$V_{out} = \frac{\sum_{i=1}^{9} C_i \times V_i}{\sum_{i=1}^{9} C_i + C_0} + 0.04\ V = \frac{(7\times 0.86\ nF + 0.1\ nF)\times 1\ V + 1\times 0.1 nF \times (-8)\ V}{7\times 0.86\ nF + 2\times 0.1\ nF + 10\ nF} + 0.04\ V$$

$$= 0.368\ V$$

Other patterns follow the same calculation method. All simulation calculated values are listed in the Supplementary Table 3.

3. Large-area implementation of the capacitive in-sensor tactile computing array

Taking the preprocessing of a m×n tactile input as an example, we employ a corresponding m×n capacitive in-sensor computing array. In the array, the T1 switches of each capacitive sensor unit are connected to corresponding input voltage biases ($V_{11}...V_{mn}$), while all T2 switches are interconnected together, ensuring that all capacitive sensing units are in parallel with a fixed capacitor C_0 (Supplementary Fig. 9). When a real object with m×n pixels is pressed onto this array, the m×n capacitive in-sensor computing array simultaneously detects all tactile input, which is then processed in sequential 3×3-pixel segments via changing controlled switches. Firstly, a 3×3 segment (magenta dotted region) is processed by controlling the electrical switch groups (T1, T2) of the 9 capacitive sensor units in that segment, outputting a corresponding V_{out} value. The switches of the remaining sensors unit remain in the off state and thus not participating in the computing procedure. Then, by toggling the controlled switches, an adjacent segment (green dotted region) is processed similarly. This sequential approach allows the m×n tactile input processing without sliding operations, as the entire array captures the tactile stimuli at once, and computation proceeds segment by segment via electrical switches.

On the other hand, to enable large-area implementation of the capacitive in-sensor tactile computing array, the miniaturization of individual capacitive sensor units becomes necessary. However, this physical scaling may decrease both the absolute capacitance value and sensitivity of the sensor due to the reduced contact area. These

reductions impose stringent requirements on the readout measurement system, demanding higher signal-to-noise ratios and lower parasitic capacitance. Consequently, a co-design optimization of capacitive sensing materials, sensor array and readout measurement system are essential for practical large-area implementations of capacitive tactile computing arrays.

4. Evaluation of power consumption

(1) Power consumption of peripheral circuitries in our system

The power consumed by peripheral circuitries includes switching circuitries, clock for switching circuitries and I/O ports in our system.

Power consumption of electrical switches: We used the TMUX1112 analog switch chips to implement the 19 electrical switches in the in-sensor computing system. The power consumption of these electrical switches consists of supply consumption, dynamic consumption and on-state consumption. We supplied a 3.3V power voltage to these switch chips. According to the user manual of the switch chip, the typical supply current is 5 nA, with a typical on-capacitance of 17 pF and an on-resistance of 3.7 Ω for each switch channel. Additionally, each of the switch channel was configured with a compliance current of 1 mA and a switching period of 61 ms (see Fig. 3d).

Supply consumption:

$$P_{supply} = I_{supply} \times V_{DD} = 5\ nA \times 3.3\ V$$
$$= 16.5\ nW$$

Dynamic consumption:

$$P_{dynamic} = C_{on} \times V_{DD}^2 \times f_{switching} = 17\ pF \times (3.3\ V)^2 \times \frac{1}{61\ ms}$$
$$= 3.09\ nW$$

On-state consumption:

$$P_{on} = I_{on}^2 \times R_{on} = (1\ mA)^2 \times 3.7\ \Omega$$
$$= 3.7\ \mu W$$

In the system, the 19 channels of the five TMUX1112 analog switch chips were used

as 19 electrical switches.

$$P_{switches} = P_{supply} \times 5 + (P_{dynamic} + P_{on}) \times 19 = 82.5\ nW + (3.09\ nW + 3.7\ \mu W) \times 19$$
$$\approx 70.4\ \mu W$$

Power consumption of logic module for switching circuitries: To provide pulse control for the three sets of electrical switches in the system, we designed a switch control logic using Verilog and implemented it on an FPGA. The resultant power consumption of the switch control logic, includes specific I/O ports and the clock for switching circuitries, was evaluated on Vivado tool based on xczu15eg-ffvb1156-2-i device (Compared to the Quartus tool, the Vivado tool can provide more details on the power consumption of I/O ports, clock and logic). The power consumption of I/O ports and clock for switching circuitries is approximately 418.7 µW, as follows:

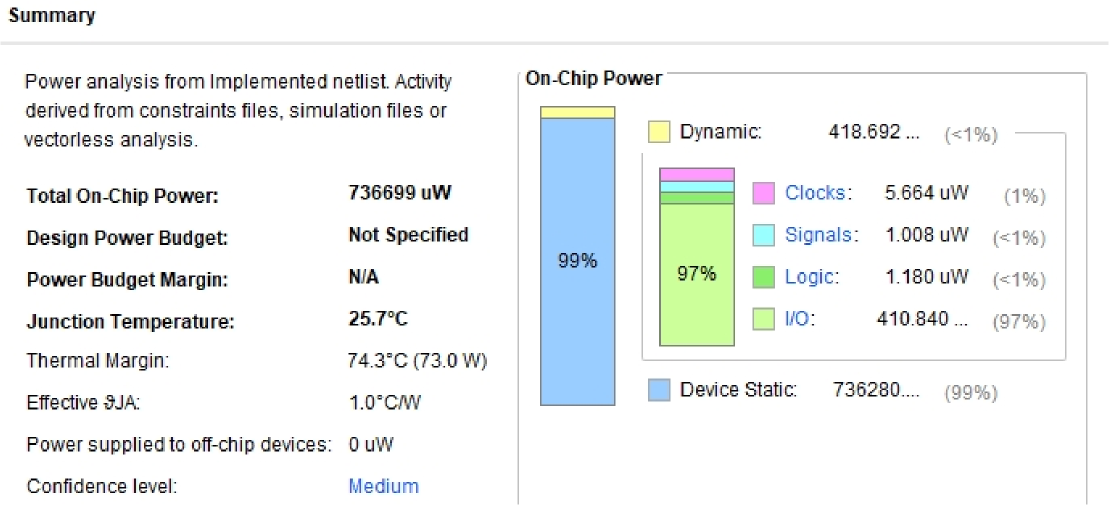

The power consumed by the capacitive sensors and fixed capacitor C_0 are 3.74 µW and 2.58 µW for the noise reduction and edge detection tasks (see Methods).

Total power consumption:

In noise reduction task, the total power consumption of the system is:

$$P_{total} = P_{switches} + P_{IO/clock} + P_{sensors} = 70.4\,\mu W + 418.7\,\mu W + 3.74\,\mu W$$

$$\approx 493\,\mu W$$

In edge detection task, the total power consumption of the system is:

$$P_{total} = P_{switches} + P_{IO/clock} + P_{sensors} = 70.4\,\mu W + 418.7\,\mu W + 2.58\,\mu W$$

$$\approx 492\,\mu W$$

(2) **Power consumption of conventional mixed electronic system**

We used the Vivado tool to estimate the total power consumption of conventional mixed electronic system. The static power consumption represents the power consumed by the FPGA core, while the dynamic power consumption refers to the I/O ports, clock and logic. Therefore, the power consumption of the conventional mixed electronic system is approximately 11154 μW.

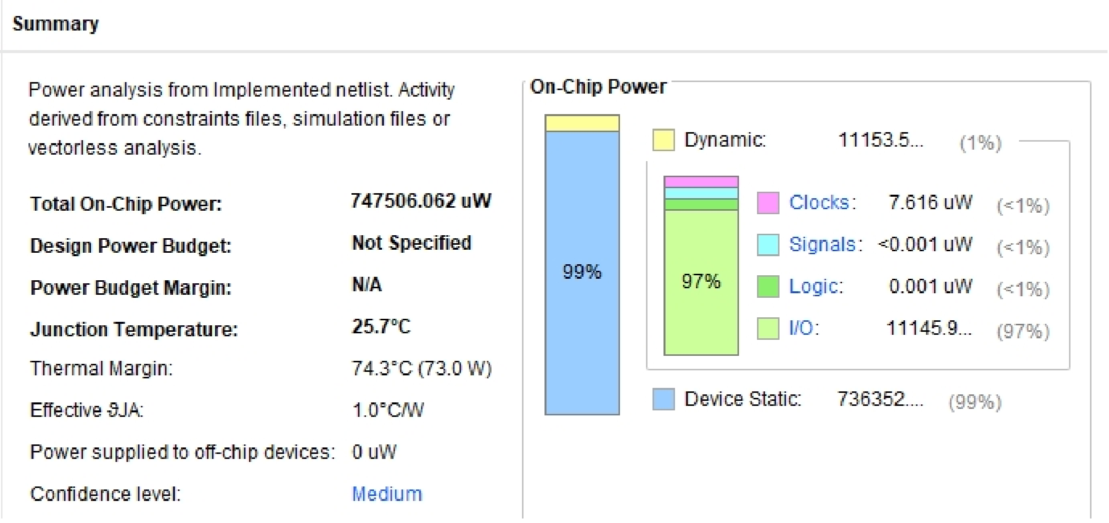

Behavioral models of the ADC and MAC

The behavioral models of the ADC and MAC are described in Verilog HDL as follows:

```
//Top entity
module mac_top (
input wire clk,
```

```
input wire rst_n,
input wire adc_en,
input wire [7:0] VA1,
input wire [7:0] VA2,
⋮
input wire [7:0] VA9,
input wire [7:0] V1,
input wire [7:0] V2,
⋮
input wire [7:0] V9,
output wire [31:0] pixel_out,
output wire valid_out
);

reg valid_in;
wire ack_1,ack_2,ack_3,ack_4,ack_5,ack_6,ack_7,ack_8,ack_9;
reg [71:0] pixel_in;
reg [71:0] voltage_in;
wire [7:0] CD1,CD2,CD3,CD4,CD5,CD6,CD7,CD8,CD9;

reg clk_10k;
reg [31:0] cnt;
always @(posedge clk or negedge rst_n)
begin
if (~rst_n)   begin
cnt <= 0;
clk_10k<=0;
end
else if (cnt == 32'd15) begin
cnt <= 0;
clk_10k<=~clk_10k;
end
else
cnt <= cnt +1;
end
//ninth instantiated adc module
adc u_adc1 (
.rst_n(rst_n),
.adc_en(adc_en),
.analog_in(VA1),
.ack_out(ack_1),
```

```
.digital_out(CD1)
);
⋮
adc u_adc9 (
.rst_n(rst_n),
.adc_en(adc_en),
.analog_in(VA1),
.ack_out(ack_1),
.digital_out(CD1)
);

mac u_mac(
.clk(clk_10k),
.rst_n(rst_n),
.valid_in(valid_in),
.voltage_in(voltage_in),
.pixel_in(pixel_in),
.pixel_out(pixel_out),
.valid_out(valid_out)
);
always @(posedge clk_10k or negedge rst_n) begin
if (!rst_n) begin
valid_in <=0;
pixel_in[71:0]<=0;
voltage_in[71:0]<=0;
end else        begin
valid_in <= ack_1 & ack_2 & ack_3 & ack_4 & ack_5 & ack_6 & ack_7 & ack_8& ack_9;
pixel_in[71:0]<={CD9,CD8,CD7,CD6,CD5,CD4,CD3,CD2,CD1};
voltage_in[71:0]<={V9,V8,V7,V6,V5,V4,V3,V2,V1};
end
end
endmodule

```

```
// ADC module
module adc (
input wire clk,
input wire rst_n,
input wire [7:0] analog_in,
input wire adc_en,
```

```
output reg    ack_out,
output reg [7:0] digital_out
);
localparam IDLE = 2'b00;
localparam CONVERTING = 2'b01;
localparam DONE = 2'b10;
reg [1:0]        state;
reg [7:0] counter;

localparam VREF = 8'hFF;

always @(posedge clk or negedge rst_n) begin
if (~rst_n)   begin
state <= IDLE;
digital_out <= 8'b0;
ack_out <= 1'b0;
counter <= 8'b0;
end else begin
case (state)
IDLE: begin
ack_out <= 1'b0;
if (adc_en) begin
state <= CONVERTING;
counter <= 8'b10000000;
digital_out <= 8'b0;
end
end
CONVERTING: begin
if (counter == 0) begin
state <= DONE;
ack_out <= 1'b1;
end else begin
if (analog_in >= digital_out + counter) begin
digital_out <= digital_out + counter;
end
counter <= counter >> 1;
end
end
DONE: begin
if (!adc_en) begin
state <= IDLE;
```

```
end
end
endcase
end
end
endmodule
```

```
// MAC module
module mac(
input wire clk,
input wire rst_n,
input wire valid_in,
input wire [71:0] voltage_in,
input wire [71:0] pixel_in,
output reg [31:0] pixel_out,
output reg valid_out
);
localparam IDLE = 2'b00;
localparam MULTIPLY = 2'b01;
localparam ACCUMULATE=2'b10;
localparam DONE = 2'b11;
reg   [1:0]   next_state;
reg    [1:0]    current_state;
reg [15:0] product [0:8];
reg [31:0] sum;

always @(posedge clk or negedge rst_n) begin
if (~rst_n) begin
current_state <= IDLE;
end else begin
current_state <= next_state;
end
end

always @(*) begin
next_state = current_state;
case (current_state)
IDLE: begin
if (valid_in) begin
next_state = MULTIPLY;
end
```

```
end
MULTIPLY: begin
next_state = ACCUMULATE;
end
ACCUMULATE: begin
next_state = DONE;
end
DONE: begin
next_state = IDLE;
end
default: next_state = IDLE;
endcase
end

always @(posedge clk) begin
if (current_state == MULTIPLY) begin
product[0] <= pixel_in[7:0]     * voltage_in[7:0];
product[1] <= pixel_in[15:8]   * voltage_in[15:8];
product[2] <= pixel_in[23:16] * voltage_in[23:16];
product[3] <= pixel_in[31:24] * voltage_in[31:24];
product[4] <= pixel_in[39:32] * voltage_in[39:32];
product[5] <= pixel_in[47:40] * voltage_in[47:40];
product[6] <= pixel_in[55:48] * voltage_in[55:48];
product[7] <= pixel_in[63:56] * voltage_in[63:56];
product[8] <= pixel_in[71:64] * voltage_in[71:64];
end
end

always @(posedge clk) begin
if (~rst_n) begin
sum <= 32'b0;
end else if (current_state == ACCUMULATE) begin
sum <= product[0] + product[1] + product[2] +
product[3] + product[4] + product[5] +
product[6] + product[7] + product[8];
end
end

always @(posedge clk) begin
if (~rst_n) begin
valid_out <= 1'b0;
end else if (current_state == DONE) begin
```

```
valid_out <= 1'b1;
end else begin
valid_out <= 1'b0;
end
end

always @(posedge clk) begin
if (current_state == DONE) begin
pixel_out <= sum;
end
end
endmodule
```

II: Supplementary Figures and Captions

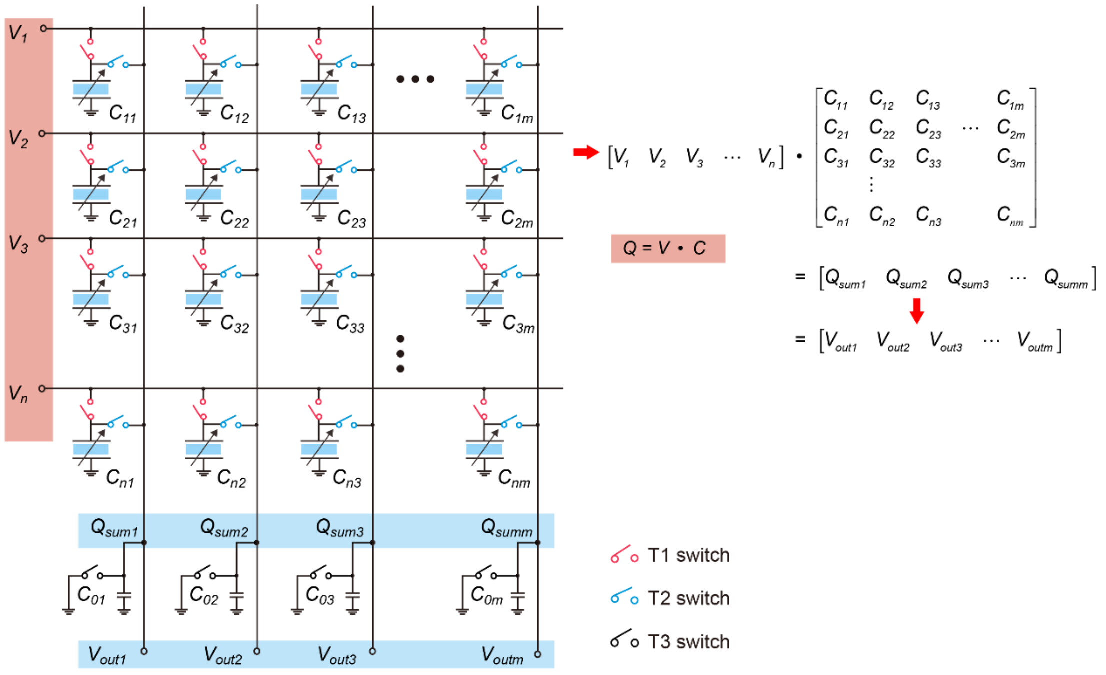

Supplementary Figure 1 Implementation of multiplication and accumulation (MAC) operations in parallel in the capacitive in-sensor tactile computing system. The input voltage (V_1, V_2, ···, V_n) is regarded as a row vector, and the capacitance value carrying pressure information (C_{11}, C_{12}, ···, C_{nm}) acts as column vectors. The dot production of one row and one column vectors can be achieved through the accumulated charges along the column, which represents a specific kind of computation functionality. All resultant accumulated electric charges (Q_{sum1}, Q_{sum2}, ···, Q_{summ}) stored on the fixed capacitor (C_{01}, C_{02}, ···, C_{0m}) along the m columns can be achieved in parallel.

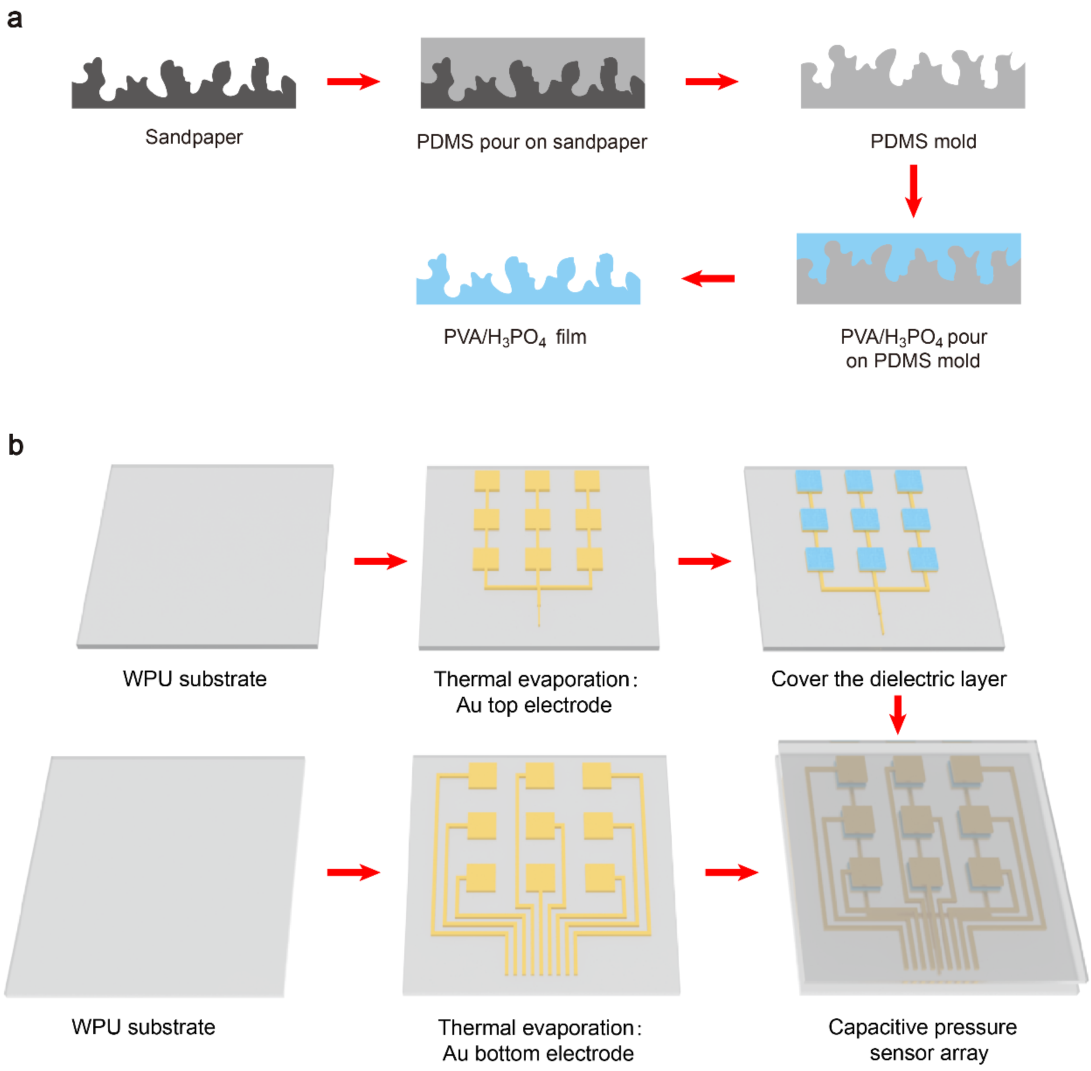

Supplementary Figure 2 Fabrication processes of the capacitive pressure sensor array. **a,** Fabrication processes of the ionic elastomeric PVA/H_3PO_4 film. **b,** Fabrication processes of the capacitive pressure sensor array by vertically stacking the patterned Au bottom electrode, microstructured PVA/H_3PO_4 sensing film, and patterned Au top electrode.

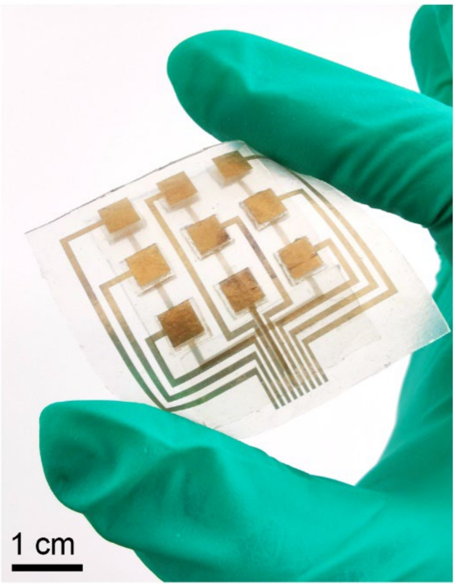

Supplementary Figure 3 Optical image of the fabricated flexible capacitive pressure sensor array.

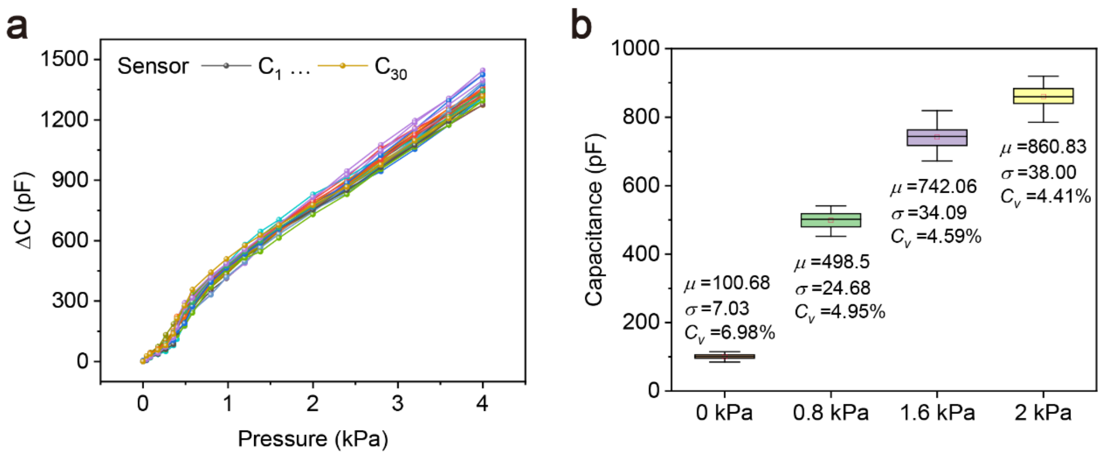

Supplementary Figure 4 **a,** Capacitance-pressure response of 30 sensor devices. The applied pressure ranges from 0 to 4 kPa. **b,** Variation coefficient of sensor under the pressure of 0, 0.8, 1.6 and 2 kPa.

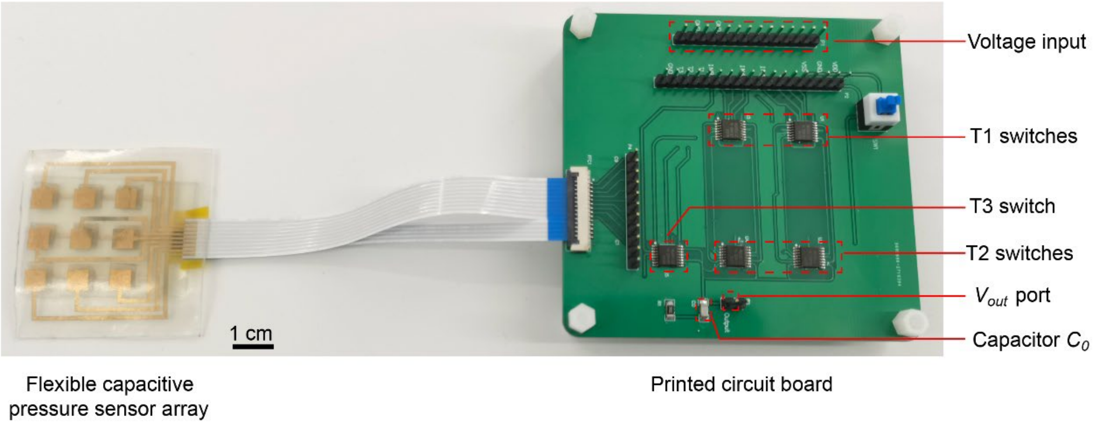

Supplementary Figure 5 Optical image of the capacitive in-sensor tactile computing system. The system consists of nine adjacent capacitive pressure sensor pixels (C_1 to C_9) with their corresponding electrical switches (T1, T2 and T3). We employed switching chips to control the on and off states of the switches T1, T2 and T3 through pulse signals. A fixed capacitor C_0 is integrated on a printed circuit board to read out the calculated result of the capacitive in-sensor tactile computing system.

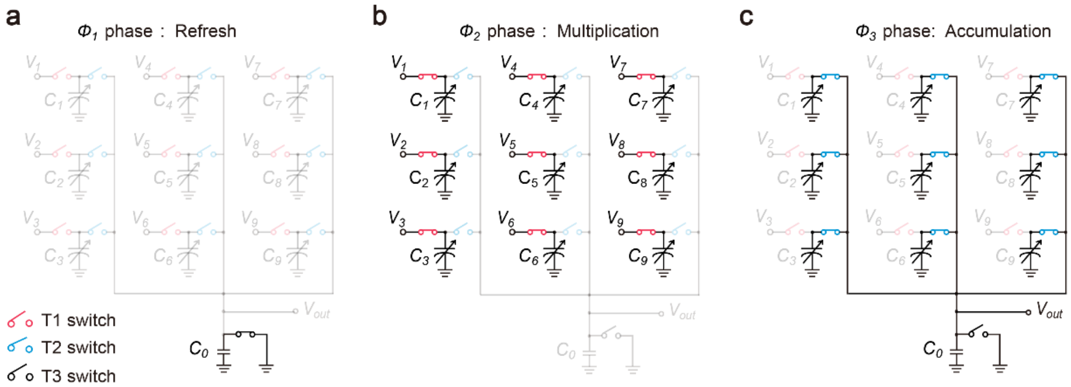

Supplementary Figure 6 Operation flow of capacitive in-sensor computing. **a,** Φ_1 phase: T3 switch turns on, while T1 switches and T2 switches turn off, the charge on C_0 is reset to zero. **b,** Φ_2 phase: T1 switches turn on, while T2 switches and T3 switch turn off, implementing nine multiplication operations. **c,** Φ_3 phase: T2 switches turn on, while T1 switches and T3 switch turn off, implementing an accumulation operation.

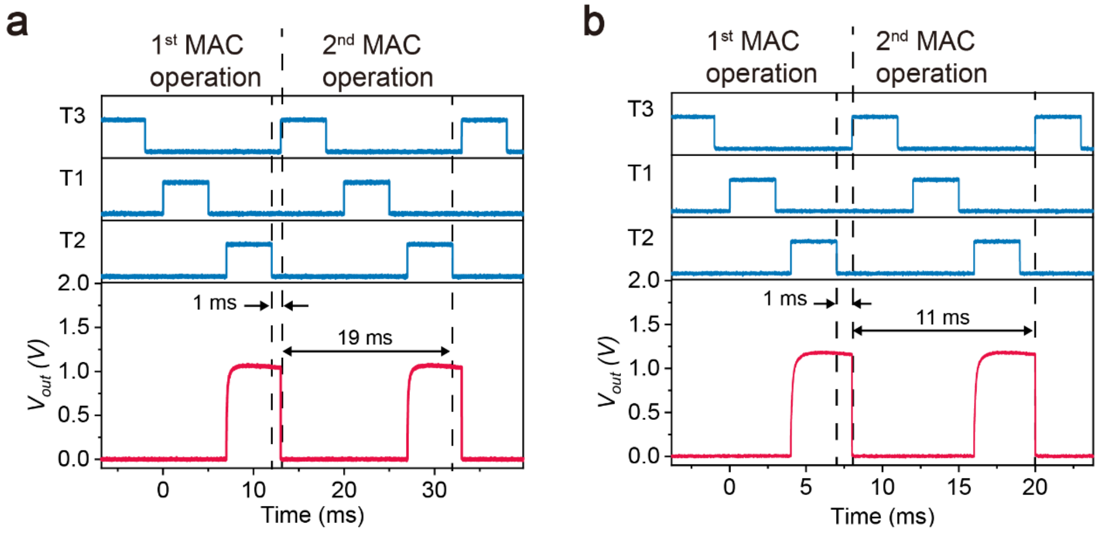

Supplementary Figure 7 Timing diagram of the capacitive in-sensor computing kernel to implement the MAC operation. **a**, The interval time between adjacent MAC operations can actually be reduced to 1 ms. **b**, Both the on-time of the electrical switches and the interval during MAC operations can be shortened.

Bullet-shaped pattern

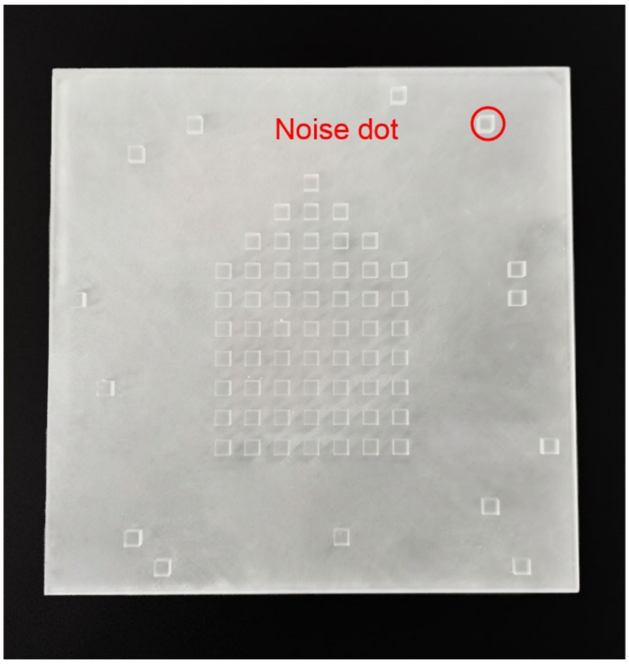

Supplementary Figure 8 Optical image of the noisy bullet-shaped mold fabricated by three-dimensional (3D) printing technology. The size of mold is 17 × 17 pixels and the distance of two adjacent pixels is 5 mm.

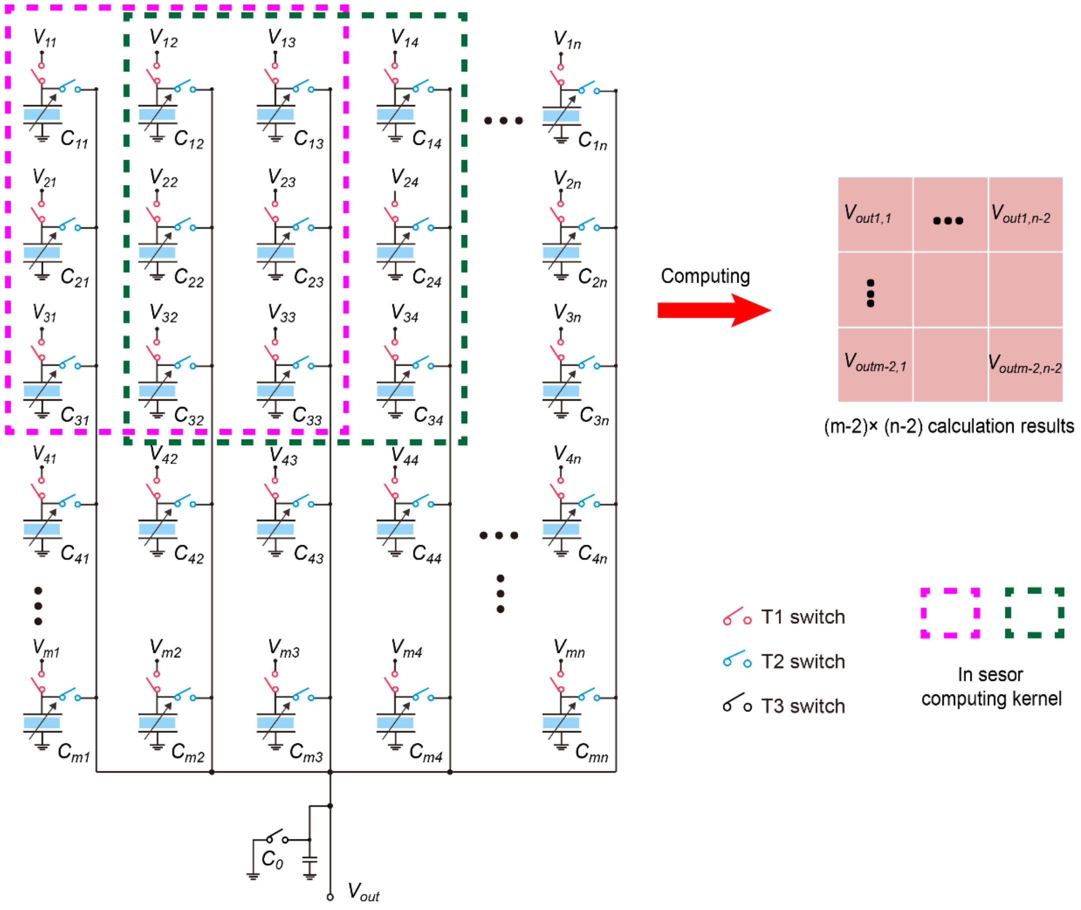

Supplementary Figure 9 Circuit schematic of the m×n capacitive in-sensor tactile computing for large-area application.

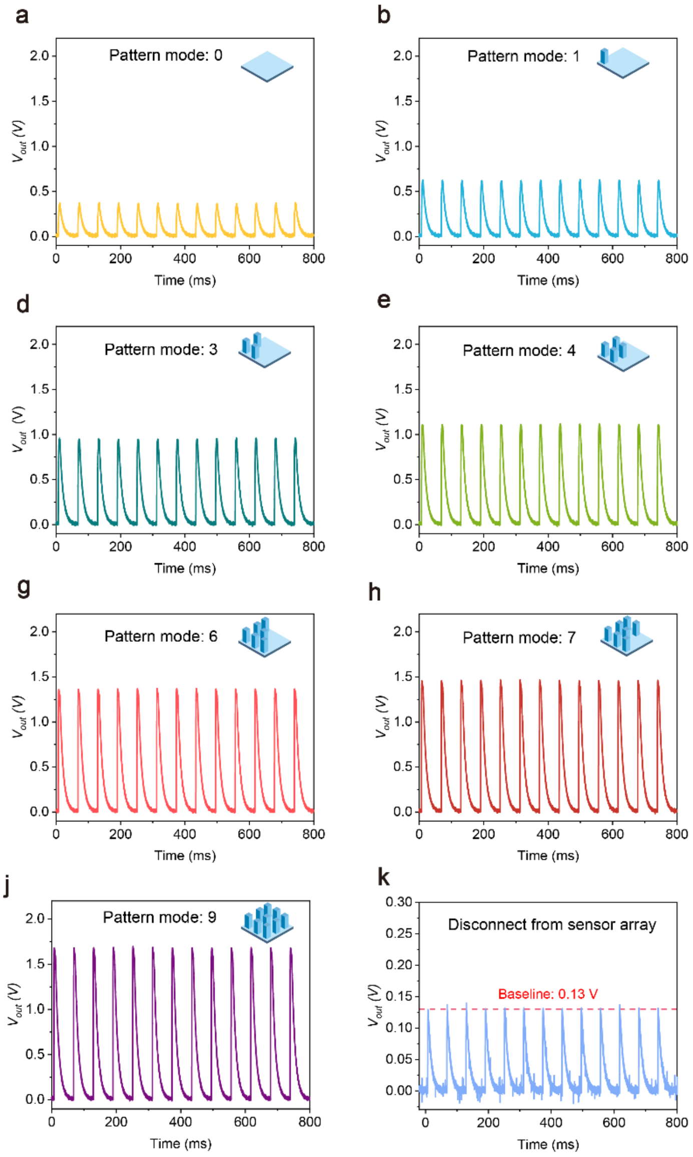

Supplementary Figure 10 **a-j,** Readout voltages of the in-sensor tactile averaging computing kernel on the capacitor C_0 for all the tactile stimulus patterns from mode 0 to mode 9. A blue pillar indicates a pressure stimulus of 2 kPa in the pressure sensor pixel, while its absence corresponds to a stimulus of 0 kPa. **k,** The baseline voltage of the system for the noise reduction task, due to the leakage current and parasitic capacitance. The baseline voltage is 0.13 V when no capacitive pressure sensor array is not connected.

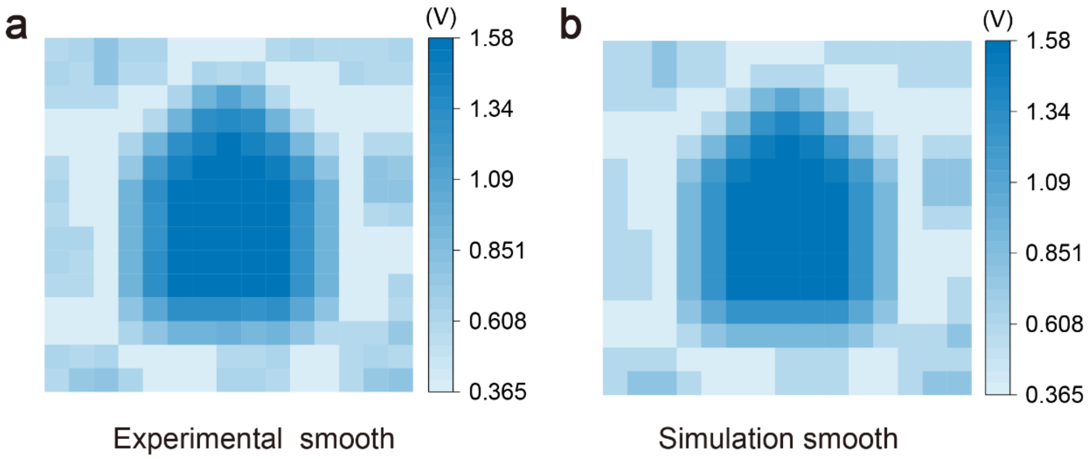

Supplementary Figure 11 a-b, Noise reduction results of the noisy bullet-shaped mold from experiment (**a**) and simulation (**b**).

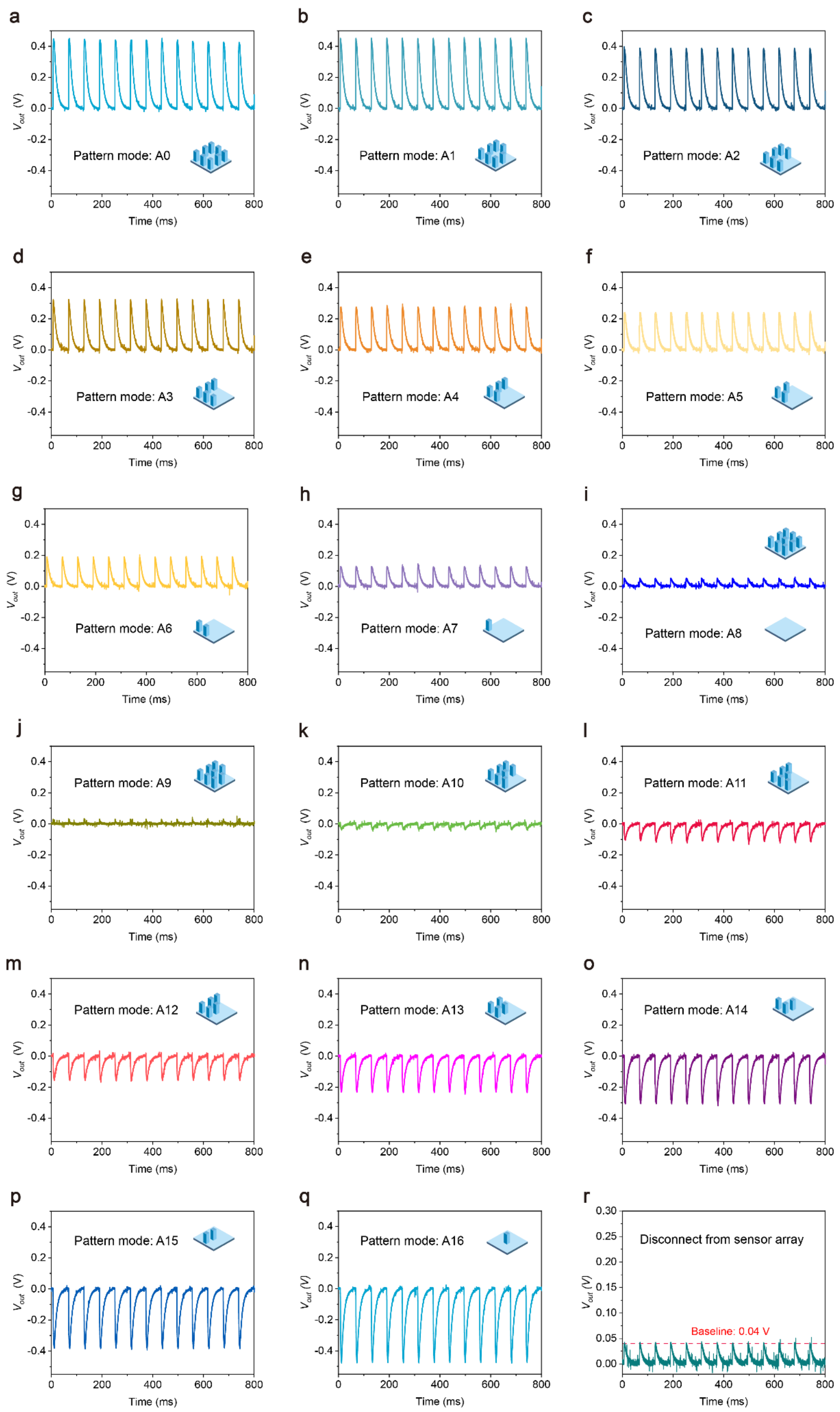
a
Pattern mode: A0
b
Pattern mode: A1
c
Pattern mode: A2
d
Pattern mode: A3
e
Pattern mode: A4
f
Pattern mode: A5
g
Pattern mode: A6
h
Pattern mode: A7
i
Pattern mode: A8
j
Pattern mode: A9
k
Pattern mode: A10
l
Pattern mode: A11
m
Pattern mode: A12
n
Pattern mode: A13
o
Pattern mode: A14
p
Pattern mode: A15
q
Pattern mode: A16
r
Disconnect from sensor array
Baseline: 0.04 V
V_{out} (V)
Time (ms)

Supplementary Figure 12 **a-q**, Readout voltages of the in-sensor tactile computing kernel on the capacitor C_0 for all the tactile stimulus patterns from mode A0 to mode A16. Here, the in-sensor computing kernel is configured as a Laplace filter. A blue pillar indicates a pressure stimulus of 2 kPa in the pressure sensor pixel, while its absence corresponds to a stimulus of 0 kPa. **r,** The baseline voltage of the system for the edge detection task, due to the leakage current and parasitic capacitance. The baseline voltage is 0.04 V when no capacitive pressure sensor array is not connected.

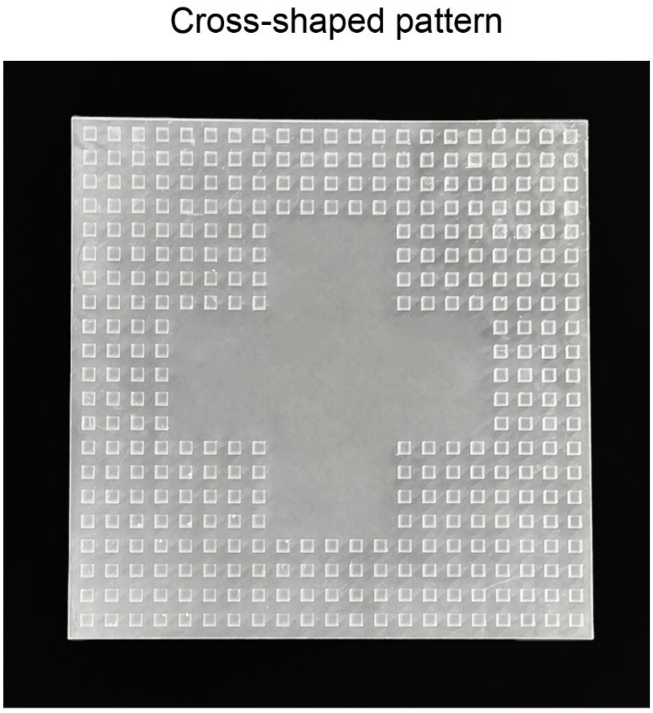

Supplementary Figure 13 Optical image of the cross-shaped object fabricated by 3D printing technology. The size of mold is 21 × 21 pixels and the distance of two adjacent pixels is 5 mm.

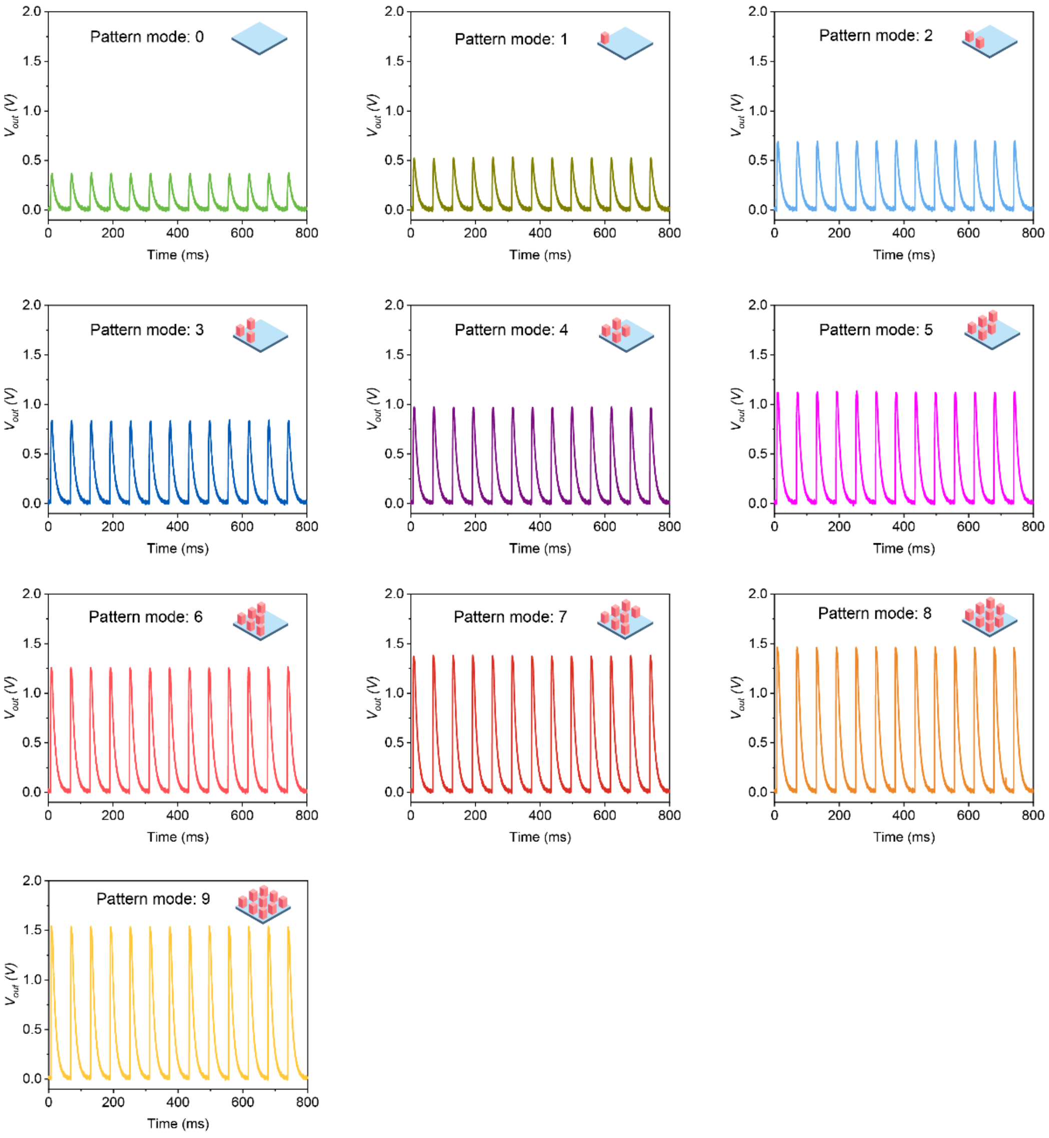

Supplementary Figure 14 **a-j**, Readout voltages on the capacitor C_0 for all the tactile stimulus patterns when only the pressure stimuli of 1.6 kPa are applied onto the sensor array. A red pillar indicates a pressure stimulus of 1.6 kPa in the pressure sensor pixel, while its absence corresponds to a stimulus of 0 kPa.

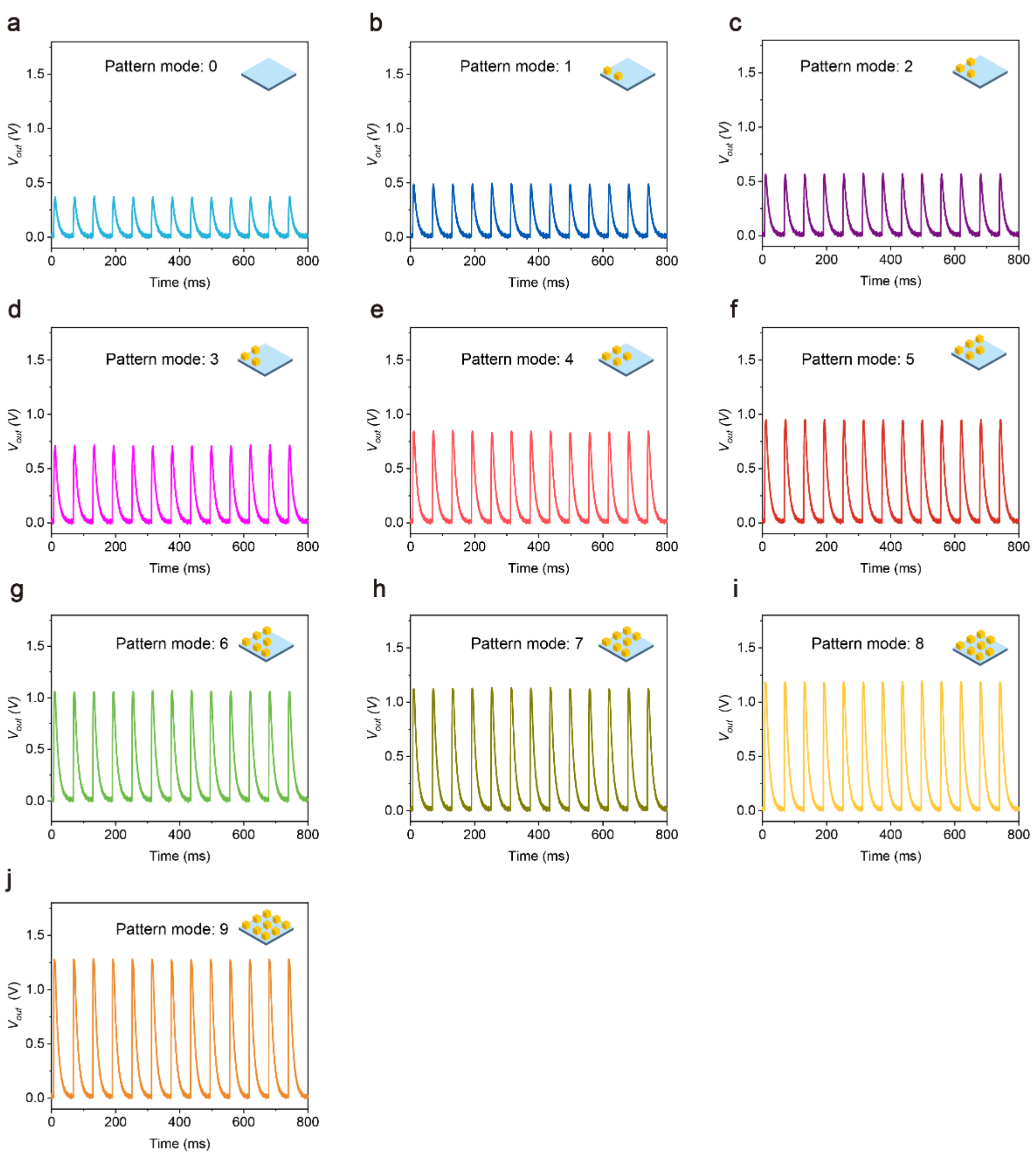

Supplementary Figure 15 **a-j**, Readout voltages on the capacitor C_0 for all the tactile stimulus patterns when only the pressure stimuli of 0.8 kPa are applied onto the sensor array. A yellow pillar indicates a pressure stimulus of 0.8 kPa in the pressure sensor pixel, while its absence corresponds to a stimulus of 0 kPa.

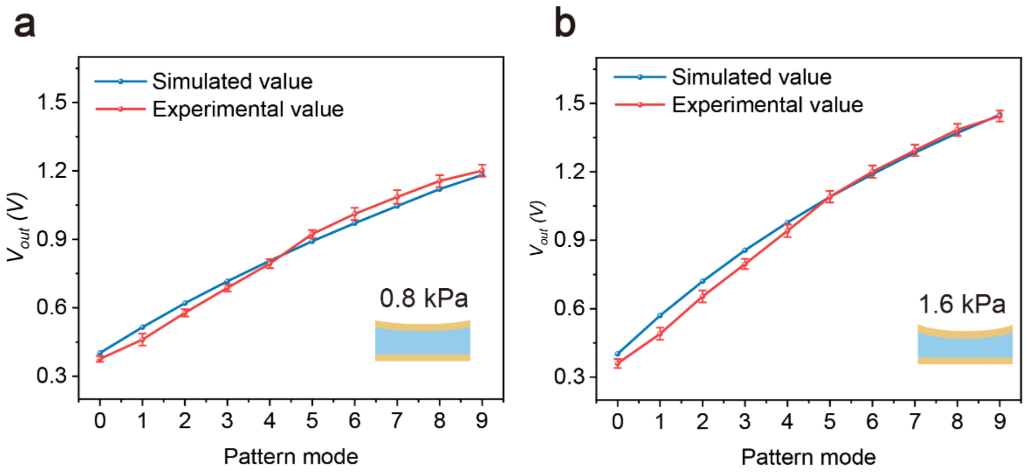

Supplementary Figure 16 a-b, Experimental and simulation calculated V_{out} for all tactile stimulus patterns when the pressure stimuli of 0.8 (**a**) and 1.6 kPa (**b**) are applied onto the sensor array, respectively. The experimental V_{out} values match with the simulated values well.

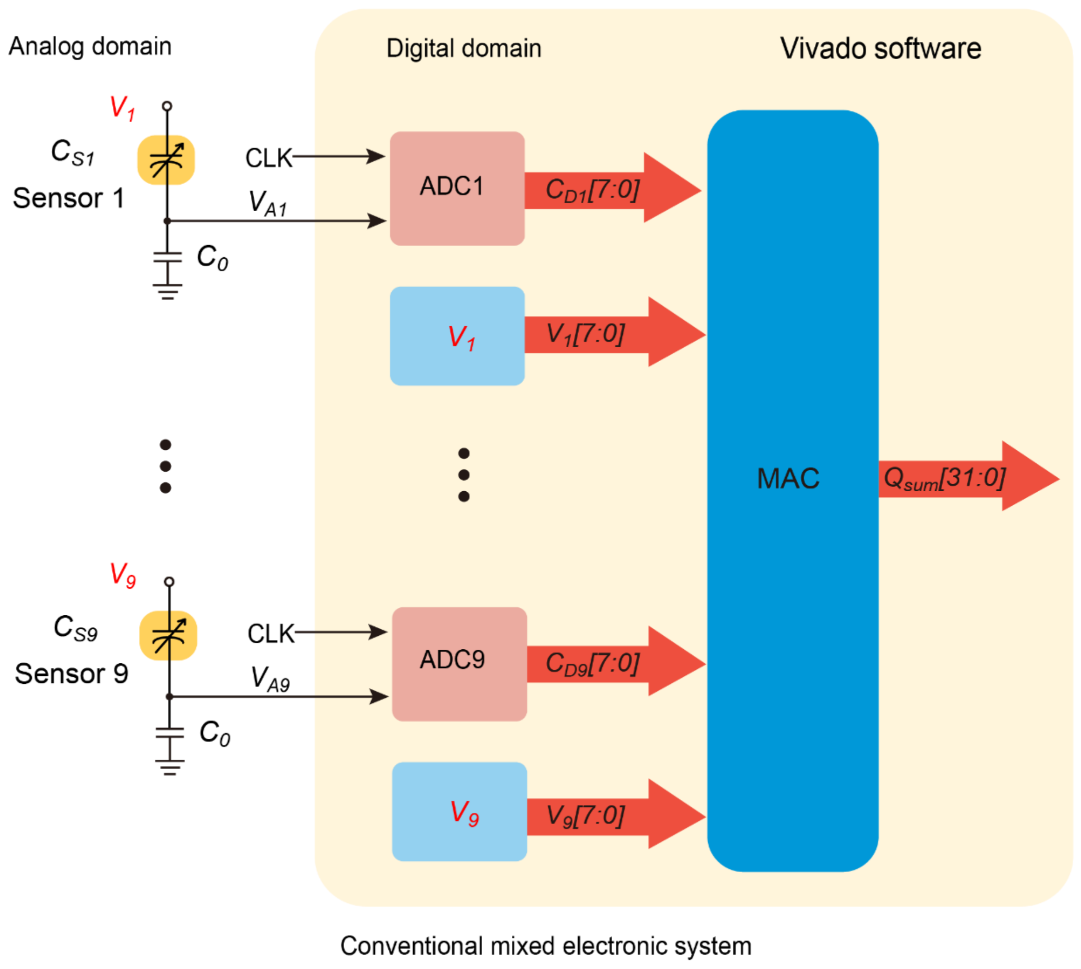

Supplementary Figure 17 Schematic diagram of a conventional mixed electronic system. The system can emulate the sensing-computing functionality similar to our capacitive in-sensor computing system. The system consists of nine parallel branches and a multiply-accumulate (MAC) module. Each branch includes a capacitive sensor, a fixed capacitor and an 8-bit analogue-to-digital converters (ADCs). When the input voltage bias of the i_{th} capacitive pressure sensor (i=1,2…n) is set to V_i, the capacitive sensor C_{Si} converts an external tactile stimulus into an analog voltage V_{Ai}. Subsequently, the analog voltage V_{Ai} is digitized by the ADC into an 8-bit digital value C_{Di}[7:0]. This digital output C_{Di}[7:0] and corresponding 8-bit digital input voltage V_i[7:0] are sent to the MAC module to perform the MAC operation, generating a 32-bit

Q_{sum}[31:0] as the output result. The ADC and MAC modules were developed as behavioral circuit models by Verilog on Vivado software to evaluate the power consumption of the conventional mixed electronic system.

III: Supplementary Tables

Supplementary Table 1 Comparison between our work and other in-sensor and near-sensor tactile computing systems.

Type	Device design		Computing architecture	Computing functionality	Functional reconfiguration	Reference
	Sensing unit	Computing unit				
Multimode-fused spiking neuron	Pressure sensor (Pressure), Memristor (Temperature)	Mott Memristor	Near/In-sensor computing	Fusing signals (spike encoding)	No	Ref 32
Crossmodal sensory neuron	Pressure sensor (Pressure), Memristor (Temperature)	Mott Memristor	Near/In-sensor computing	Fusing signals (spike encoding)	No	Ref 31
Calibratable sensory neuron	Pressure sensor (Pressure), Light sensor (Light), Memristor (Temperature)	Mott Memristor	Near-sensor computing	Fusing signals (spike encoding)	No	Ref 25
Artificial tactile near-sensor computing unit	Triboelectric sensor	Synaptic transistor	Near-sensor computing	Threshold detection	No	Ref 23
Tactile near-sensor analogue computing	Pressure sensor	Nonvolatile memristor	Near-sensor computing	Vector-matrix multiplication	Yes (Averaging filter, Laplacian filter)	Ref 26
IR touch sensor	Pressure sensor		In-sensor computing	Fusing signals	No	Ref 33
LPI tactile sensor	Pressure sensor		In-sensor computing	Fusing signals	No	Ref 34
Capacitive in-sensor tactile computing	Pressure sensor		In-sensor computing	Vector-matrix multiplication	Yes (Averaging filter, Laplacian filter)	This work

Supplementary Table 2 Simulation calculated values with the in-sensor tactile computing kernel as the average filter under the pressure of 2 kPa.

Pattern mode	V_{out} (V)
0	0.402
1	0.600
2	0.773
3	0.926
4	1.063
5	1.185
6	1.295
7	1.395
8	1.487
9	1.57

Supplementary Table 3. Simulation calculated values when the in-sensor tactile computing kernel as the Laplace filter under the pressure of 2 kPa.

Pattern mode	V_{out} (V)
A0	0.398
A1	0.368
A2	0.335
A3	0.299
A4	0.258
A5	0.213
A6	0.162
A7	0.105
A8	0.04
A9	-0.005
A10	-0.054
A11	-0.107
A12	-0.167
A13	-0.233
A14	-0.306
A15	-0.388
A16	-0.481
